# Discovery of a neuromuscular syndrome caused by biallelic variants in *ASCC3*

**DOI:** 10.1016/j.xhgg.2021.100024

**Published:** 2021-01-21

**Authors:** Divya Nair, Dong Li, Hannah Erdogan, Andrew Yoon, Margaret H. Harr, Gaber Bergant, Borut Peterlin, Maruša Škrjanec Pušenjak, Parul Jayakar, Rolph Pfundt, Sandra Jansen, Kirsty McWalter, Alpa Sidhu, Sheila Saliganan, Emanuele Agolini, Arthur Jacob, Jennifer Pasquier, Rafii Arash, Kimia Kahrizi, Hossein Najmabadi, Hans-Hilger Ropers, Elizabeth J. Bhoj

**Affiliations:** 1 Children’s Hospital of Philadelphia, Philadephia, PA, USA; 2 Clinical Institute of Genomic Medicine, University Medical Centre Ljubljana, Ljubljana, Slovenia; 3 Nicklaus Children’s Hospital, Miami, FL, USA; 4 Department of Human Genetics, Donders Institute for Brain, Cognition and Behaviour, Radboudumc, the Netherlands; 5 GeneDx, Gaithersburg, MD, USA; 6 University of Iowa, Iowa City, IA, USA; 7 Ambry Genetics, Aliso Viejo, CA, USA; 8 Translational Cytogenomics Research Unit, Bambino Gesù Children's Hospital, IRCCS, Rome, Italy; 9 Department of Genetic Medicine, Genetic Intelligence Laboratory, Weill Cornell Medicine in Qatar, Education City, Al Luqta St, Ar-Rayyan, Qatar; 10 Genetics Research Center University of Social Welfare and Rehabilitation Sciences, Tehran, Iran; 11 Max Planck Institute for Molecular GeneticsIhnestr. 63-73, Berlin, Germany

**Keywords:** Activating Signal Cointegrator 1 Complex, Subunit 3, ASCC3, neurogenetics, neuromuscular

## Abstract

Activating Signal Cointegrator 1 Complex, Subunit 3 (ASCC3) is part of the four-part ASC-1 transcriptional cointegrator complex. This complex includes ASCC1 (associated with spinal muscular atrophy with congenital bone fractures 2), TRIP4 (associated with spinal muscular atrophy with congenital bone fractures 1), and ASCC2 (not yet associated with human disease.) *ASCC3* encodes a DNA helicase responsible for generating single-stranded DNA as part of the DNA damage response. Interestingly, *ASCC3* expresses coding and non-coding isoforms, which act in opposition to balance the recovery of gene transcription after UV-induced DNA damage. Here we report the discovery of *ASCC3* as the cause of a neuromuscular syndrome in seven unreported individuals from six unrelated families and updates on the one previously reported family. All the individuals share a neurologic phenotype that ranges from severe developmental delay to muscle fatigue. There appears to be genotype-phenotype correlation, as the most mildly affected individual is homozygous for a rare missense variant, while the more severely affected individuals are compound heterozygotes for a missense and a presumed loss-of-function (LOF) variant. There are no individuals with biallelic presumed LOF variants in our cohort or in gnomAD, as this genotype may not be compatible with life. In summary we report a syndrome in these eleven individuals from seven families with biallelic variants in *ASCC3*.

## Teaser

Although other members of the ASC-1 transcriptional cointegrator complex are associated with severe neuromuscular syndromes, the phenotype of *Activating Signal Cointegrator 1 Complex, Subunit 3* (*ASCC3*) has not previously been delineated. Here we describe 11 individuals with a variable pediatric-onset neuromuscular syndrome caused by biallelic variants in *ASCC3*.

## Main text

The Activating Signal Cointegrator 1 (ASC-1) complex is a transcriptional cointegrator complex that plays an important role in gene transcription.^1^ Within the ASC-1 complex are four subunits, Activating Signal Cointegrator 1 Complex (ASCC)-1, -2, and -3, as well as Thyroid Hormone Receptor Interactor 4 (TRIP4). Biallelic loss-of-function (LOF) pathogenic variants in *TRIP4* and *ASCC1* cause spinal muscular atrophy with congenital bone fractures (SMABF) 1 (MIM: 616866) and 2 (MIM: 616867). SMABF was first described as a unique entity in 1991.^2^ It has a significant overlap with the classic *SMN1*-related spinal muscular atrophy (MIM: 253300). The SMABF phenotype is dominated by very severe early-onset neuromuscular disease. Affected individuals often have fetal hypokinesia resulting in arthrogryposis multiplex congenita and prenatal long-bone fractures. The neuromuscular issues these individuals experience are typically severe enough to cause death from respiratory failure.^3^ Homozygous LOF variants in *TRIP4* have also been linked to one family with Davignon-Chauveau type congenital muscular dystrophy (MIM: 617066), a less-severe form of the disease.^4^ Functional studies of LOF variants in *TRIP4* demonstrated the vital role of ASC-1 in the regulation of skeletal myogenesis.^5^ ASCC2, also a member of the complex, has not yet been linked to human disease. The ASC-1 complex in general has also been functionally linked to amyotrophic lateral sclerosis (ALS). RNAP II/U1 small nuclear ribonucleoprotein particle (snRNP) machinery, responsible for Mendelian forms of ALS, inappropriately dissociates from the ASC-1 complex with pathogenic SMABF variants, demonstrating an interesting link between the two disorders.^6^

Functionally, ASCC3 is a DNA helicase that generates single-stranded DNA during the DNA damage response cascade. It was recently reported that *ASCC3* expresses both a coding and non-coding isoform. Williamson et al.^7^ showed that these two isoforms have a cross-talk mechanism that allows for a rheostat action for ramping gene transcription up or down based on UV-induced DNA damage. Cells that lack the short isoform of ASCC3 are unable to restart transcription after UV damage, and those that lack the long isoform have inappropriately increased transcription after UV damage.

**Figure 1:**
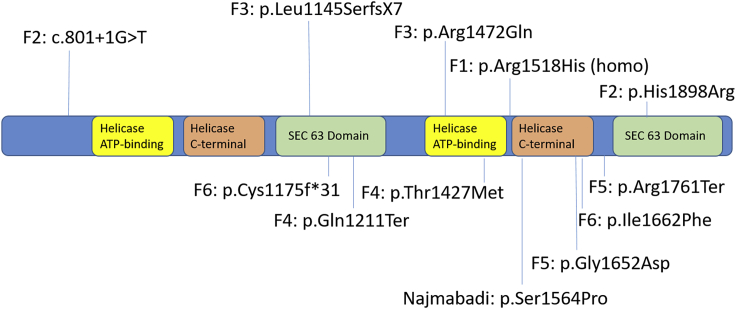
Functional consequences of variants in the ASCC3 protein Variants include missense, splice site, and loss-of-function variants.

Homozygous *ASCC3* variants have been previously reported in only one family with intellectual disability by Najmabadi et al.^8^ in 2011. This study centers on intellectual disability gene discovery, and they briefly report four family members with homozygous missense *ASCC3* variants identified by exome sequencing that segregate with the phenotype. The unaffected parents are first cousins, and the affected individuals are reported to exhibit mild non-syndromic intellectual disability. We provide additional clinical information as well as unpublished clinical photos of these four adult siblings. In addition, we report seven individuals from six unrelated families, who have not been previously reported, with biallelic variants in *ASCC3* (Figure 1; Table S1; Supplemental notes). Although there is a range in severity of disease, they share a neuromuscular phenotype that is generally less severe than SMABF (Table 1).

**Table 1:** Summary of clinical findings in unreported individuals with biallelic *ASCC3* variants

	No. of individuals (%)
Developmental delay	6/6 (100%), range mild to severe
Hypotonia	5/6 (83%)
Large central incisors	3/6 (50%)
Abnormal palate	3/6 (50%)
Feeding difficulties	3/6 (50%)
Severe constipation	2/6 (33%)
Extreme fatigue	2/6 (33%)
Hypomimic face	2/6 (33%)
Low-set ears	2/6 (33%)
Upturned nose	2/6 (33%)
Thin upper lip	2/6 (33%)
IUGR	2/7 (29%)

Developmental delay, hypotonia, large central incisors, abnormal palate, and feeding difficulty were described in at least half of the children. Clinical details of the six live-born individuals (three females and four males aged prenatal to 11 years) are included.

The Institutional Review Board of the Children’s Hospital of Philadelphia approved this study. Informed consent was obtained from all individual participants included in the study. Additional informed consent was obtained from all individual participants for whom identifying information is included in this article. Genomic DNA was extracted from whole blood from the affected children and their parents. Exome or genome sequencing was performed with a variety of standard capture kits and the general assertion criteria for variant classification following ACMGG/AMP guidelines. There were no other variants in these individuals that remained after filtration and analysis using either dominant or recessive models and could explain the phenotypes. The families were grouped for this study through personal communication and GeneMatcher.^9^ The published article includes all datasets generated or analyzed during this study.

**Figure 2:**
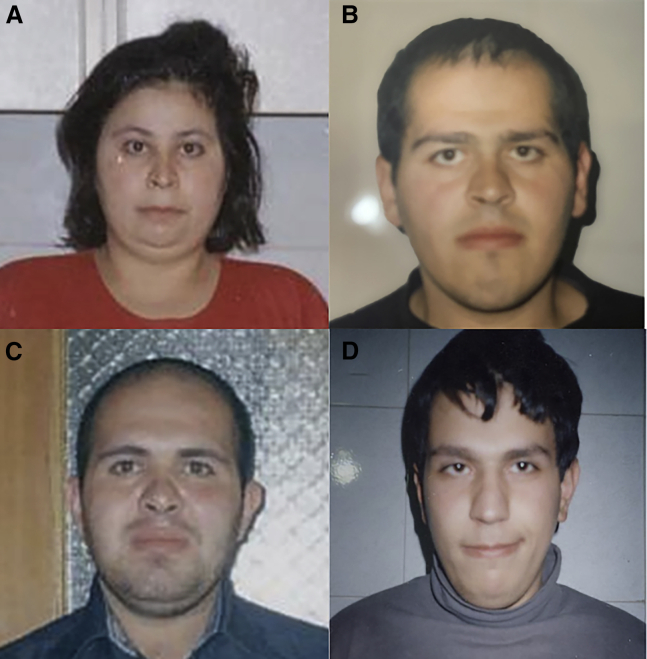
Facial features of four siblings with biallelic *ASCC3* variants Individuals (A) 7-1, (B) 7-2, (C) 7-3, and (D) 7-4 (previously reported by Najmabadi et al.,^8^ but without photographs). They do not have distinctly dysmorphic features but share bushy eyebrows with prognathism.

These eleven individuals from seven unrelated families all have biallelic variants in *ASCC3* and a variable neurobehavioral and neuromuscular phenotype All ten of the post-natal individuals had some reported developmental delays, and 5/10 had significant hypotonia. Brain malformations were reported in one fetus (cerebral and cerebellar hypoplasia) and one infant (cerebellar lobe hypoplasia). Only one individual had developmental regression, consistent with autistic regression, suggesting that this is not necessarily a progressive disorder. Dysmorphic facial features that were reported in at least two families included low-set/posteriorly rotated ears, large front teeth, upturned nose, and thin upper lip; however, only one family consented for publication of photographs (Figure 2). Neither major congenital anomalies nor seizures appear to be a significant part of the phenotype. It is likely that this disorder will continue to be diagnosed through exome sequencing and other unbiased testing, as it is clinically ambiguous.

As hypotonia and fatigue are the most significant and common feature among six of the seven families, the differential diagnosis would include spinal muscular atrophy, congenital myopathies, metabolic myopathies, or muscular dystrophies. Based on these individuals, we suggest that when a clinician encounters a cluster of symptoms including global developmental delay, hypotonia, and/or fatigue, but without other major anomalies, then biallelic variants in *ASCC3* should be considered. As with any autosomal recessive disease, there should be a higher level of concern if consanguinity is reported. However, five of these seven families have compound heterozygous variants; this may suggest that biallelic presumed LOF variants are prenatally lethal.

After a diagnosis of *ASCC3*-related myopathy, clinicians should consider a skeletal survey and DEXA scan, neurodevelopmental and feeding evaluation, echocardiogram, sleep study, and auditory testing. Referring for early intervention including speech, occupational, and physical therapy is especially recommended in these individuals, as they will be critical to maximize their developmental potential.

As expected, the phenotypes of these individuals overlap with those previously reported individuals with SMABF who have variants in the genes that encode the other proteins in the ASC-1 complex, *ASCC1* and *TRIP4*. Those individuals have much more severe neuromuscular disease with fragile bones. Our individuals are generally more mildly affected than SMABF individuals, and only 1/10 individuals have significant bone findings, which are similar to those with SMABF. Our families may be more mildly affected, as they are all either compound heterozygous for a combination of a presumed LOF and missense variant (5/7) or homozygous for a missense variant (2/7) in the least affected individuals. Interestingly we did not find any individuals, either in our affected cohort or in gnomAD, with biallelic presumed LOF variants, which suggests this may not be compatible with postnatal life.

In conclusion, we present a neuromuscular syndrome caused by biallelic variants in *ASCC3* in eleven individuals from seven unrelated families. Individuals with biallelic missense variants may be especially likely to remain under-diagnosed during exome or genome sequencing, as these variants are particularly difficult to interpret. Therefore, it is especially important to focus on any low-frequency alleles found in *ASCC3* in individuals with a matching neuromuscular phenotype.

## Supplemental information

Document S1. Supplemental notes

Table S1. Molecular, demographic, and clinical data for seven previously unreported and four previously reported individuals with biallelic *ASCC3* variants Developmental delay, hypotonia, large central incisors, abnormal palate, and feeding difficulty were described in at least half of the children. (Hom: Homozygous, Combined Annotation Dependent Depletion: CADD, -: Absent, +: Present, NR: Not reported, NP: Not performed)

Document S2. Article plus supplemental information
