# Discovery of a neuromuscular syndrome caused by biallelic variants in *ASCC3*

**DOI:** 10.1016/j.xhgg.2022.100122

**Published:** 2022-07-12

**Authors:** Divya Nair, Dong Li, Hannah Erdogan, Andrew Yoon, Margaret H. Harr, Gaber Bergant, Borut Peterlin, Maruša Škrjanec Pušenjak, Parul Jayakar, Rolph Pfundt, Sandra Jansen, Kirsty McWalter, Alpa Sidhu, Sheila Saliganan, Emanuele Agolini, Arthur Jacob, Jennifer Pasquier, Rafii Arash, Kimia Kahrizi, Hossein Najmabadi, Hans-Hilger Ropers, Elizabeth J. Bhoj

## Main text

(Human Genetics and Genomics Advances *2*, 100024; April 8, 2021)

Patients 3-1 and 3-2 (siblings) were tested at GeneDx. They were compound heterozygous for p.Arg1472Glu and c.3434del p.Lys1145fs∗7. However c.3434del p.Lys1145fs∗7 was noted as p.Leu1145fs∗7 in Table S1 and the supplemental text. This has now been corrected, and the authors regret the error.

